# The Effect of Blood-Derived Products on the Chondrogenic and Osteogenic Differentiation Potential of Adipose-Derived Mesenchymal Stem Cells Originated from Three Different Locations

**DOI:** 10.1155/2019/1358267

**Published:** 2019-12-31

**Authors:** Markus Neubauer, Olga Kuten, Christoph Stotter, Karina Kramer, Andrea De Luna, Thomas Muellner, Zsombor Lacza, Stefan Nehrer

**Affiliations:** ^1^Department of Orthopedics, University Clinic Krems, Mitterweg 10, 3500 Krems, Austria; ^2^Danube University Krems, Center for Regenerative Medicine and Orthopedics, Dr. Karl-Dorrek-Str. 30, A-3500 Krems, Austria; ^3^Department for Orthopedics and Traumatology, Landesklinikum Baden-Mödling, Waltersdorfer Str. 75, 2500 Baden, Austria; ^4^Department of Orthopedics and Traumatology, Evangelic Hospital Vienna, Hans-Sachs-Gasse 10–12 1180 Vienna, Austria; ^5^University of Physical Education, Alkotás u. 44, Budapest, Hungary H-1123

## Abstract

**Background:**

Adipose-derived mesenchymal stem cells (AD-MSCs) from fat tissue considered “surgical waste” during joint surgery may provide a potent source for regenerative medicine. Intra-articular, homologous fat tissue (Hoffa's fat pad, pouch fat) might possess a superior chondrogenic and osteogenic differentiation potential in comparison to extra-articular, nonhomologous fat. Blood products might further enhance this potential.

**Methods:**

AD-MSCs were isolated from fat tissue of 3 donors from 3 locations each, during total knee replacement. Isolated cells were analyzed via flow cytometry. Cells were supplemented with blood products: two types of platelet-rich plasma (EPRP—PRP prepared in the presence of EDTA; CPRP—PRP prepared in the presence of citrate), hyperacute serum (hypACT), and standard fetal calf serum (FCS) as a positive control. The viability of the cells was determined by XTT assay, and the progress of differentiation was tested via histological staining and monitoring of specific gene expression.

**Results:**

Blood products enhance ex vivo cell metabolism. Chondrogenesis is enhanced by EDTA-PRP and osteogenesis by citrate PRP, whereas hyperacute serum enhances both differentiations comparably. This finding was consistent in histological analysis as well as in gene expression. Lower blood product concentrations and shorter differentiation periods lead to superior histological results for chondrogenesis. Both PRP types had a different biological effect depending upon concentration, whereas hyperacute serum seemed to have a more consistent effect, independent of the used concentration.

**Conclusion:**

(i) Blood product preparation method, (ii) type of anticoagulant, (iii) differentiation time, and (iv) blood product concentration have a significant influence on stem cell viability and the differentiation potential, favouring no use of anticoagulation, shorter differentiation time, and lower blood product concentrations. Cell-free blood products like hyperacute serum may be considered as an alternative supplementation in regenerative medicine, especially for stem cell therapies.

## 1. Introduction

Chondral and osteochondral lesions progress to joint degeneration, lead to osteoarthritis, and contribute to the potential necessity for TJR [1, 2]. Regenerative orthopedics aims for joint preservation and articular cartilage regeneration in order to delay or fully avoid TJR.

Due to the physiological architecture of articular cartilage, without vessel- or nerve endings, its intrinsic regenerative capacity is limited [3, 4]. This clinical need leads to the ongoing development of therapies to regenerate hyaline cartilage.

Autologous chondrocyte transplantation is a profoundly studied treatment approach to meet this demand [5–7]. Nevertheless, its limitations such as the necessity for a two-step surgical procedure or the dedifferentiation potential of ex vivo-cultured chondrocytes *drive* the development of novel, preferably one-step procedures [8].

MSCs have been in the focus of research over the course of the past years for regenerative and joint preservative applications mainly due to their (i) differentiation potential into chondrogenic tissue—amongst others such as fat and osteogenic tissue—as well as the (ii) *modulatory* features of MSCs' secretome consisting of growth factors, cytokines, and extracellular vesicles [9, 10].

MSCs exist in various tissues such as bone marrow or adipose tissue [11, 12]. BMA is a traditional MSC harvest site due to the minimal cell manipulation necessary as well as the possibility for a “point-of-care” application [13]. AD-MSCs provide the same advantages while elegantly skipping the considerable comorbidity of BMA harvest. This additional value gave rise to extensive interest in clinical applications of AD-MSCs.

**Figure 1:**
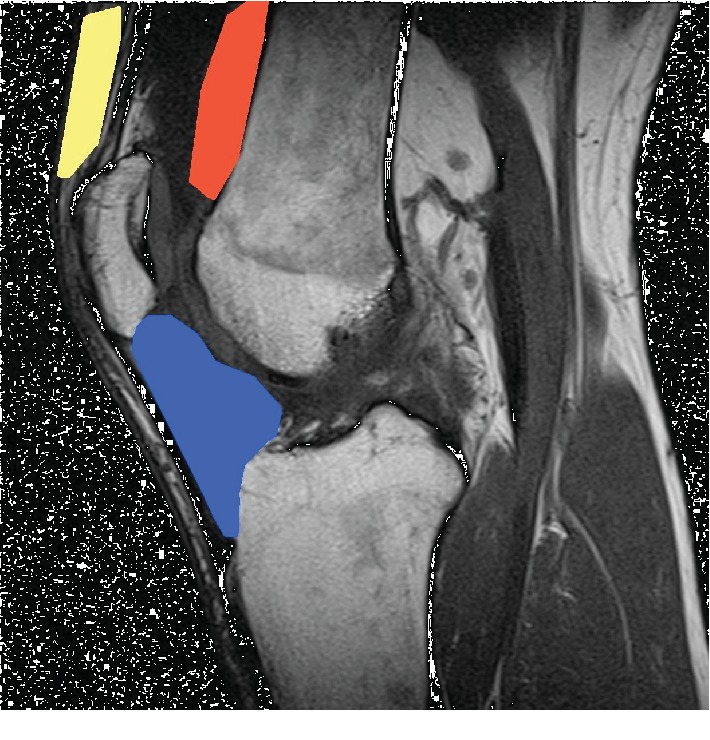
Sagittal knee MRI with periarticular fat sources (yellow—subcutaneous fat; red—prefemoral/supratrochlear pouch fat; blue—infrapatellar fat pad/Hoffa's fat pad).

During arthroscopic or open-knee surgery, various fat sources likely yielding AD-MSCs are accessible (Figure 1). The subcutaneous fat lies directly under the skin, is easily accessible and usually available in high quantity (yellow). The prefemoral fat pad (supratrochlear pouch) lies on the anterior aspect of the femur, just above the trochlea (red). Furthermore, the infrapatellar fat pad (also known as Hoffa's fat pad) is located within the knee joint and fills the space behind the patellar tendon between the patella, femoral condyles, and the tibia plateau (blue). The infrapatellar fat pad can be visualized and accessed during arthroscopic surgery. Utilizing adipose tissue in this manner surpasses the need for liposuction and thus eliminates the risk of its associated comorbidities [14, 15].

The first clinical trials back in 2011 combined AD-MSCs with autologous blood-derived products in order to further increase the likelihood of therapeutic effects [16, 17]. The rationale for blood product supplementation was to enhance stem cell growth in the joint [18, 19].

Blood products have recently become a widely used treatment in regenerative medicine [20]. The underlying rationale is to separate blood components by centrifugation that are likely to be therapeutically useful—platelets, fibrin, growth factors, etc.—from those that are not (e.g., erythrocytes) [20, 21]. Best known and widely used is the PRP. However, PRP's heterogeneous production protocols make the comparability difficult. Different compositions may target specific biological processes demonstrating the necessity for an extensive understanding of the underlying biologic mechanisms of action. PRP was also reported to contain proinflammatory components such as leucocytes or fibrin [22, 23]. hypACT is a newly developed blood product without cells and fibrin containing the cytokines released during blood clotting [24] which can serve as an alternative to PRP's products.

Regulatory hurdles regarding the use of “nonhomologous tissue” such as “transplanting” components of subcutaneous adipose tissue into joints make these promising strategies increasingly challenging [25].

Preclinical data indicate that blood-derived products support the maintenance of cell proliferation, as well as stem cell properties and differentiation characteristics in in vitro settings [26, 27]. Moreover, a combination of platelet lysate and human plasma may reduce senescence of AD-MSCs in culture [27]. PRP-enriched medium specifically was shown to enhance chondrogenic differentiation of AD-MSCs [28].

Moreover, literature provides data showing better chondrogenic differentiation potential of intra-articular “homologous” adipose tissue such as the Hoffa's fat pad in comparison with subcutaneous tissue AD-MSCs demonstrating the importance of the harvest location choice [29–31]. Despite a rich body of preclinical data investigating the effects of blood products as well as the effects of location upon the regenerative and differentiation potential of AD-MSCs, a paucity of studies investigate a potential synergistic effect of versatile combinations of those two. This gap in knowledge was central to conceptualize this study.

Clinical data dealing with adipose-derived stem cells and their effects upon cartilage regeneration do not focus on comparing AD-MSCs from different locations but only investigate subcutaneous tissue as a source [17, 18]. Likewise, blood product-supplemented AD-MSC applications are not well represented despite studies indicating a potential superiority of these supplementations also in the joint [17, 18, 32, 33]. Sound in vitro data are needed to (i) demonstrate the benefits of the synergistic use of blood product supplementation alongside with harvest location choice in order to (ii) help design optimized treatment regimes for clinical trials taking harvest location and ideal blood product supplementation into account.

The rationale for this study was that homologous AD-MSCs in adipose tissue considered “surgical waste” during arthroscopy or open-knee surgery may provide an autologous source for regenerative use. This mode of application would surpass regulatory hurdles, use homologous tissue with potentially superior differentiation potential, and may be further improved by blood product supplementation.

**Figure 2:**
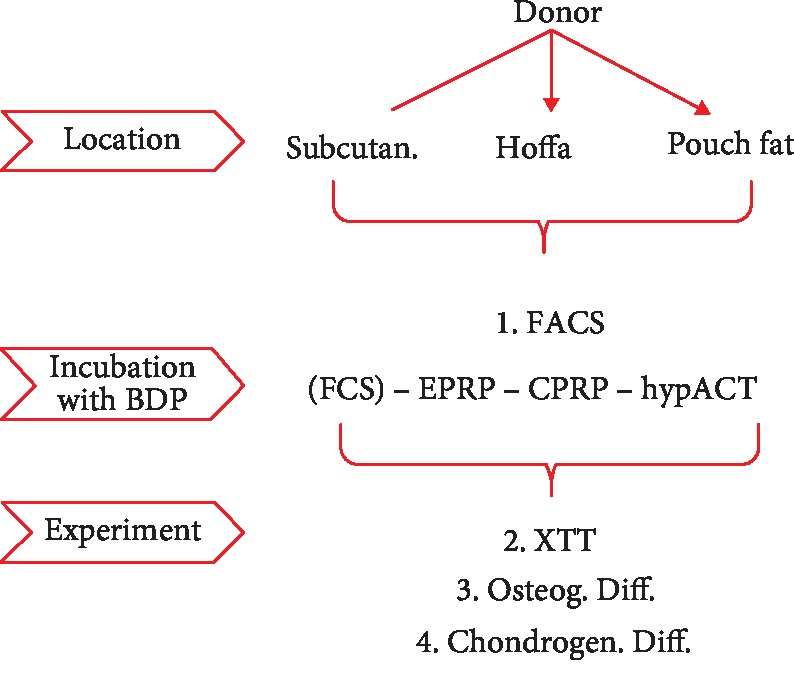
Study design. BDP=blood-derived products; chondrogen.=chondrogenic; CPRP=citrate platelet-rich plasma; EPRP=EDTA platelete-rich plasma; FACS=fluorescence-activated cell sorting; FCS=fetal calve serum; hypACT=hyperacute serum; osteogeny.=osteogenic; subcutan.=subcutaneous; XTT=assay for metabolic activity.

The aim of this study was to (i) compare chondrogenic and osteogenic differentiation of AD-MSCs from 3 fat locations (Hoffa's fat pad, distal femur/pouch fat, and subcutaneous fat) and to (ii) demonstrate effects of three blood products (EPRP, CPRP, and hypACT) on the differentiation potential in an in vitro setting (Figure 2).

## 2. Materials and Methods

### 2.1. Cell Harvest

Adipose tissue was harvested from 3 donors (1 male, 2 female, mean age ± SD = 71.3 ± 2.9 years) from 3 locations each (2 intra-articular tissues: Hoffa's fat pad, distal femur/pouch fat; 1 extra-articular tissue: subcutaneous fat) during TJR, resulting in 9 groups. The tissue was stored at +8° in a PBS solution. One out of three donors received intra-articular knee injections containing corticosteroids (Diprophos**®** (Betamethason) 0.5 ml = 1/2 vial diluted in 1 ml = 1 vial of Xyloneural® (Lidocaine)) whereas the other two donors received intra-articular injections containing pure hyaluronan (Hialurom® (Hyaluronate 30 mg/2 ml)). Written informed consent was obtained from all subjects. This study was approved by the Ethics Committee of the Evangelic Hospital Vienna (Positive Vote: 03.05.2018).

### 2.2. Isolation and Cell Culture

Adipose tissue was minced with a scalpel, weighted, and digested in a collagenase (catalogue number: C0130, Sigma-Aldrich, St. Louis, USA) solution (9000 units of collagenase I/15 ml DMEM/10 g of fat) for 2 hours at 37°C. Digestion was followed by filtration (Corning® 40 *μ*m Cell Strainer, catalogue number: 431750, Durham, USA) and neutralization with standard MSC growing media (DMEM, high glucose, GlutaMAX™ Supplement, pyruvate, catalogue number: 31966047, Gibco Life Technologies Europe Bv, Bleiswijk, Netherlands) with the addition of the antibiotics 2% Penicillin/Streptomycin (catalogue number: P4333, Sigma-Aldrich Chemie GmbH, Steinheim, Germany) and 1% Amphotericin B (catalogue number: A2942, Sigma-Aldrich Chemie GmbH, Steinheim, Germany), 10% fetal calf serum (catalogue number: 11550356, Gibco™, qualified, heat inactivated, Gibco Life Technologies Europe Bv, Bleiswijk, Netherlands), 1% nonessential amino acids (MEM NEAA catalogue number: 11140050, Gibco Life Technologies Europe Bv, Bleiswijk, Netherlands), and bFGF 1 ng/ml (Fibroblast Growth Factor-Basic, human, catalogue number: F0291, Sigma-Aldrich Chemie GmbH, Steinheim, Germany). After the following centrifugation (700 × g for 10 min), the supernatant was removed and the remaining cells were seeded at a density of 10,000 cells/cm^2^ in standard MSC growing media. Cells were cultured until reaching approximately 80-90% confluency, with nonadherent cells discarded after 24 hours and then every 2-3 days. Cells were detached with Accutase (catalogue number: SCR005, Sigma-Aldrich Chemie GmbH, Steinheim, Germany), centrifuged, and diluted in freezing media (10% DMSO (catalogue number: D8418, Sigma-Aldrich Chemie GmbH, Steinheim, Germany) and 90% FCS (catalogue number: 11550356, Gibco™)). 1 million cells in 1 ml of freezing media were collected in cryovials and stored in alcohol-free cell freezing containers in a -80°C freezer overnight. The next day samples were transferred to the liquid nitrogen tank where they were stored in the nitrogen vapour phase.

Passage 1—obtained after splitting the cells following isolation from native tissue—was then either immediately used for experiments or cryopreserved in liquid nitrogen. Consequentially, only cells from P1 or P2 were used for the experiments.

For thawing the cells, the samples were immediately transferred from liquid nitrogen to the 37°C water bath for <1 minute. Thawed samples were diluted using prewarmed standard MSC growing media and centrifuged. If the viability after thawing were >70%, cells were seeded at a density of 10,000 cells/cm^2^ in standard MSC growing media.

### 2.3. Preparation of Blood Products

#### 2.3.1. Hyperacute Serum

hypACT was prepared as described in Kardos et al. [34]. Briefly, whole blood was drawn from 8 healthy donors (25–45 years) with the hypACT device (catalogue number: 700194, OrthoSera, Krems an der Donau, Austria) according to the manufacturer's protocol. The sample was immediately centrifuged at 1710 × g for 5 min at room temperature. After centrifugation, a top (platelet-rich fibrin clot) and a bottom layer were formed. The bottom layer, primarily containing red blood cells, was removed.

#### 2.3.2. Platelet-Rich Plasma

Whole blood from the same 8 donors as in the case of the hypACT preparation, was drawn in VACUETTE 9 ml K3EDTA (catalogue number: 454217; Greiner Bio-One, Kremsmünster, Austria) and VACUETTE 9 ml 9NC Trinatriumcitrat 3.2% blood collection tubes (catalogue number: 455322; Greiner Bio-One, Kremsmünster, Austria) and centrifuged at 440 × g for 10 min at room temperature. Three layers were formed in the tube: (i) a bottom layer (red blood cells), (ii) a middle layer (with the buffy coat), and (iii) a top layer (plasma). Leukocyte-poor PRP was obtained by plasma layer aspiration without the middle layer (buffy coat). The aspirate was transferred into a 15 ml falcon tube and centrifuged again at 1700 × g for 10 min. The formed platelet pellet was resuspended in 50% of the remaining platelet-poor-plasma supernatant.

Blood products were pooled and immediately used for experiments or stored at -80°C for later use.

### 2.4. Differentiation (Osteogenic and Chondrogenic)

Osteogenic differentiation was performed in 6-well culture plates in a 2D monolayer culture and in a pellet culture system for chondrogenic differentiation. The incubation time in the standard growing medium was 2-4 days (until cells reached 90-100% confluency for osteogenesis). The differentiation process was induced by the addition of special factors and different serum supplementations: EPRP, CPRP (with the addition of 2 U/ml heparin (catalogue number: 3909969, Gilvasan GmbH, Vienna, Austria)), hyperacute serum, and FCS. The following osteogenic media were used: Gibco DMEM, high glucose, GlutaMAX™ Supplement, pyruvate (catalogue number: 10569010), 100 nM dexamethasone (catalogue number: D4902, Sigma-Aldrich Chemie GmbH, Steinheim, Germany), 50 *μ*g/ml ascorbic acid (catalogue number: A4403, Sigma-Aldrich Chemie GmbH, Steinheim, Germany), and 10 mM *β*-glycerol phosphate (catalogue number: G9422, Sigma-Aldrich Chemie GmbH, Steinheim, Germany).

The following were used for chondrogenic differentiation: Gibco DMEM, high glucose, GlutaMAX™ Supplement, pyruvate (catalogue number: 10569010, LifeTech Austria, Vienna, Austria), 1% ITS (ITS Liquid Media Supplement (100×), catalogue number: I3146, Sigma-Aldrich Chemie GmbH, Steinheim, Germany), 100 nM dexamethasone (catalogue number: D4902, Sigma-Aldrich Chemie GmbH, Steinheim, Germany), 50 *μ*g/ml ascorbic acid (catalogue number: A4403, Sigma-Aldrich Chemie GmbH, Steinheim, Germany), 1% nonessential amino acids (MEM NEAA catalogue number: 11140050, Gibco Life Technologies Europe Bv, Bleiswijk, Netherlands), 5 ng/ml TGFbeta-3 (catalogue number: AF-100-36E, PeproTech, New York, USA), and 4% methyl cellulose (Sigma-Aldrich Chemie GmbH, Steinheim, Germany) with the addition of 1% or 5% different blood product supplementation or FCS. 250,000 adipose-derived stem cells were mixed with chondrogenic media and placed in 15 ml polypropylene tubes. To form the pellets, samples were centrifuged at the highest speed of 4164 × g for 10 minutes.

The differentiation experiments lasted 3 weeks, and specific differentiation media were changed 2 times per week.

### 2.5. Metabolic Activity Assay (XTT Assay)

AD-MSCs were seeded in 96-well plates (catalogue number: 260860, Thermo Fisher Scientific, Waltham, USA) at a density of 6250 per cm^2^ (2000 cells/well) and grown for 48 h in 100 *μ*l standard growing medium. 48 hours after seeding (day 0), the standard growing medium was replaced with 100 *μ*l containing 10% of each blood product (EPRP, CPRP, and hyperacute serum) or 10% FCS as the control group. In the case of PRP supplementation, 2 U/ml heparin (catalogue number: 3909969, Gilvasan GmbH, Vienna, Austria) was added to avoid clotting. Metabolic activity of the AD-MSCs was investigated by the XTT assay (catalogue number: 11465015001, Roche Diagnostics GmbH, Mannheim, Germany) according to the manufacturer's protocol. For every treatment, 3 wells were used as a control for background absorbance (medium with/without blood product) and 5 wells were used for the measurement of absorbance for each supplementation at a specific time point. Relative absorbance was measured by using a plate reader (Synergy 2, BioTek Instruments, Inc., Vermount, USA). Absorbance was measured at 492 nm, with a reference wavelength of 690 nm. The measurement was completed on days 0, 1, 3, and 6. The values obtained after the absorbance measurement were divided by the absorbance value from day 0 to obtain the value 1 for day 0 and fold change values on days 1, 3, and 6.

### 2.6. FACS

Flow cytometry with human MSC lineage-specific markers—positive for CD73, CD105, and CD90 and negative for CD34, CD11b, CD19, CD45, and HLA-DR—was performed to demonstrate stem cell specificity besides their attachment to the plastic before differentiation experiments [35]. AD-MSCs were cultured to 90% of confluency in standard growing media. Media was changed every 3 days. Cells were detached with Accutase (catalogue number: A6964, Sigma-Aldrich Chemie GmbH, Schnelldorf, Germany), centrifuged in FACS buffer (1% (*v*/*v*) FCS in phosphate-buffered saline (PBS)), and stained with antibodies, as described in Table 1 (catalogue number: 562245, BD Stemflow hMSC Analysis Kit, BD Biosciences, St. Louis, USA). Samples were incubated with antibodies for 30 min at room temperature in darkness.

Flow cytometry analysis was performed using the FC500 Flow Cytometer (Beckman Coulter, Brea, CA, USA). The flow was performed on live cells and the negative gates were set on isotype control.

### 2.7. RNA Extraction and Quantitative Real-Time Polymerase Chain Reaction (qRT-PCR)

#### 2.7.1. RNA Extraction

After differentiation, cells were collected and pooled group wise in 200 *μ*l PBS. Cells were homogenized by adding the ceramic beads (MagNA Lyser Green Beads catalogue number: 03358941001, Roche Diagnostics, Basel, Switzerland) in a MagNA Lyser device (Roche™ MagNA Lyser Benchtop Homoginizaton System). The homogenization (6500 rpm, 20 s) was repeated twice with a 2 min cooling phase in between (8-12 C°). The RNA was isolated using the High Pure RNA Isolation Kit (catalogue number: 11828665001, Roche Diagnostics GmbH, Mannheim, Germany) according to the manufacturer's protocol. RNA was eluted and stored at -80°C until cDNA synthesis.

#### 2.7.2. Gene Expression Analysis

cDNA synthesis was performed using the Transcriptor First Strand cDNA Synthesis Kit (catalogue number: 04379012001, Roche, Basel, Switzerland). RT-qPCR was performed using the FastStart Essential DNA Probes Master Kit (catalogue number: 06402682001, Roche, Basel, Switzerland) in triplicates using the LightCycler® 96 from Roche. 1 *μ*l of the cDNA product, FastStart Probe Master 2x, hydrolysis probe (final concentration 250 nM), and primers (final concentration of 900 nM) were used for PCR amplification. Primers for each gene were designed to span introns to exclude genomic contamination in PCR products and were designed using the Universal ProbeLibrary System Assay Design. In total, eight genes were analyzed: collagen type 2 (COL2AB), collagen type 1 (COL1), SOX9, matrix metalloproteinase-3 (MMP3), alkaline phosphatase (ALPL), runt-related transcription factor 2 (RUNX2), and osteocalcin (OCN). The sequences of the used primers are presented in Table 2. GAPDH was used as a housekeeping gene. PCR conditions were optimized for annealing temperature and limited cycle number (40) to ensure that product formation was in the responsive range.

### 2.8. Histology

Chondrogenic staining was performed with Alcian Blue (catalogue number: A3157, Sigma-Aldrich Chemie GmbH, Schnelldorf, Germany), Hematoxylin (Mayer's Hematoxylin, catalogue number: S3309, DakoCytomation, Carpinteria, USA), and Eosin (catalogue number: 230251, Sigma-Aldrich Chemie GmbH, Schnelldorf, Germany).

Pellets were dried, placed on the base mold (Fisherbrand™ Disposable Base Molds, catalogue number: 22-363-554, Fisher Scientific, Hampton, USA), filled with frozen tissue matrix (Tissue-Tek® O.C.T.™, catalogue number: 4583, Sakura Finetek, Alphen aan den Rijn, The Netherlands), and transferred to -80°C for at least 24 hours. Frozen pellets were cut (6 *μ*m thickness) on the cryostat device (Cryostar NX70, Thermo Fisher Scientific, Waltham, USA) and placed on adhesive glass slides (Thermo Scientific™ SuperFrost Plus™, catalogue number: 10149870, Thermo Fisher Scientific, Waltham, USA). Slides were dried and fixed in cold acetone (-20°C) for 10 minutes.

Staining in Alcian Blue (catalogue number: A3157, Sigma-Aldrich Chemie GmbH, Schnelldorf, Germany) solution lasted 30 minutes. Sections were washed in running tap water for 1 minute and dehydrated through 95% ethanol and 2 changes of absolute ethanol, for 3 minutes each. Slides were cleared with xylene, mounted with xylene, and covered with glass. Cartilage glycosaminoglycans appeared blue.

Hematoxylin (Mayer's Hematoxylin, catalogue number: S3309, DakoCytomation, Carpinteria, USA) and Eosin (catalogue number: 230251, Sigma-Aldrich Chemie GmbH, Schnelldorf, Germany) staining was performed according to the manufacturer's protocol (DakoCytomation).

Pictures were taken with a light microscope (DM-1000 Microscope, Leica Microsystems) and processed using the Leica Manager software (Leica Microsystems, Wetzlar, Germany).

### 2.9. Osteogenic Staining with Alizarin Red

Cells were fixed with formalin (catalogue number: HT501128, Sigma-Aldrich Chemie GmbH, Schnelldorf, Germany) solution 10%, with an incubation time of 30 minutes at room temperature. The fixative solution was removed, and the samples were washed with PBS. Cells were stained with 2% Alizarin Red Stain Solution (Alizarin Red S, catalogue number: A5533, Sigma-Aldrich Chemie GmbH, Schnelldorf, Germany). 2 g of Alizarin Red was dissolved in 100 ml of distilled water, filtered, and pH was adjusted to a value of 4.3. Cells were covered with the dye solution for 30 minutes at room temperature. After removing the solution, cells were washed with water and analyzed under the microscope. Areas of mineralization appeared red. Pictures were taken with a light microscope (DM-1000 Microscope, Leica Microsystems). Images were processed using the Leica Manager software (Leica Microsystems, Wetzlar, Germany). Afterwards, the stained cells were incubated with 10% acetic acid for 30 minutes and collected with a cell scraper and vortexed. Samples were then heated at 85°C for 10 minutes, cooled down, and centrifuged at 20,000 × g for 15 minutes. The supernatant was neutralized with 10% ammonium hydroxide. The absorbance was measured at 405 nm wavelength with a plate reader (Synergy 2, BioTek Instruments, Inc., Vermount, USA).

### 2.10. Statistics

Statistical analysis was performed using GraphPad Prism five software. PCR results were analyzed by using the ANOVA test combined with the Tukey-Kramer multiple comparison posttest. Metabolic activity data were analyzed applying two-way ANOVA and Bonferroni posttest. ANOVA assumptions were verified. *p* < 0.05 was considered significant. Data are presented as mean ± SEM.

## 3. Results

### 3.1. Preparation of Blood Products

Hyperacute serum, EPRP, and CPRP were prepared from whole blood from the same 8 donors; however, these revealed different blood cell contents (Table 1). All blood products had much lower RBC and WBC concentrations compared to normal values for peripheral blood cell counts [36]. Therefore, the EPRP and CPRP are leukocyte-poor PRPs. The hyperacute serum had a very low platelet concentration, whereas PLT concentration in EPRP was approximately 3-4 times more and approximately 2 times more in CPRP versus whole blood.

### 3.2. FACS—Stem Cells Isolated from All Three Locations Express Positive MSC Markers

**Figure 3:**
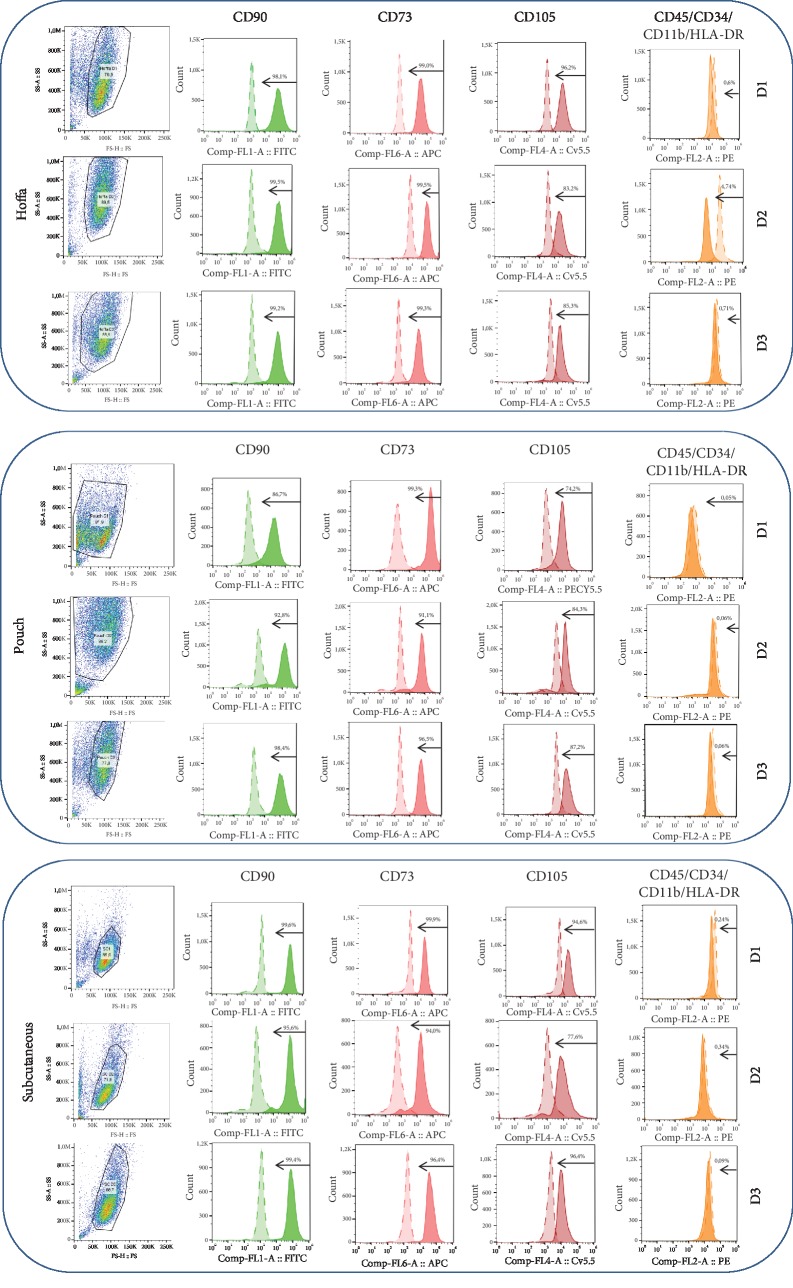
Flow cytometry results. Green=CD90; light pink=CD73; dark pink=CD105; orange=negative markers' cocktail CD45/CD34/CD11b/CD19/HLA-DR (CD=cluster of differentiation; D=donor).

Most of the isolated cells from all three locations expressed the positive mesenchymal stem cell surface markers (CD73, CD90, and CD105) [37] and did not express the markers which are typical for hematopoietic stem cells, monocyte macrophages, leukocytes, and lymphocytes (CD45, CD34, CD11b, CD19, and HLA-DR) (Figure 3, Table 3) [35]. Therefore, the isolation method was sufficient to obtain adipose stem cell populations.

### 3.3. XTT—hypACT and CPRP Enhance AD-MSC Metabolic Activity Ex Vivo

The highest increase of metabolic activity regarding fold changes was observed in both the intra-articular location groups of Hoffa's fat pad and pouch fat independent of the blood product subgroup. Subcutaneous-derived cells showed the least fold changes. The pattern of activity increase regarding fold changes per day and group was similar in both intra-articular structures compared to subcutaneous-derived cells.

The least overall-fold change in metabolic activity was observed in the EPRP subgroup consistently throughout all locations.

**Figure 4:**
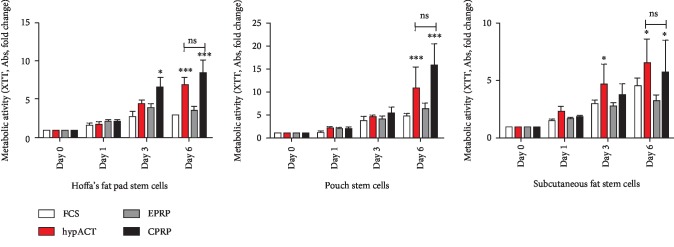
Metabolic activity. CPRP=citrate platelet-rich plasma; EPRP=EDTA platelete-rich plasma; FCS=fetal calve serum; hypACT=hyperacute serum; ns=nonsignificant. Results are expressed as the mean ± SD. ∗ represents *p* < 0.05, ∗∗ represents *p* < 0.01, and ∗∗∗ represents *p* < 0.001.

Hoffa's fat pad stem cells expressed significantly higher metabolic activity already at day 3 in the group supplemented with CPRP and with hyperacute serum in the population isolated from subcutaneous fat. At day 6, the CPRP and hypACT subgroups showed significantly greater metabolic activity compared to FCS and EPRP in all three locations (*p* < 0.05). The difference between the hypACT and CPRP subgroups on day 6 was not significant in any stem cell populations (Figure 4).

### 3.4. Histology—EPRP and hypACT Enhance Chondrogenic Differentiation Whereas CPRP and hypACT Enhanced Osteogenic Differentiation

#### 3.4.1. Chondrogenic Differentiation

**Figure 5:**
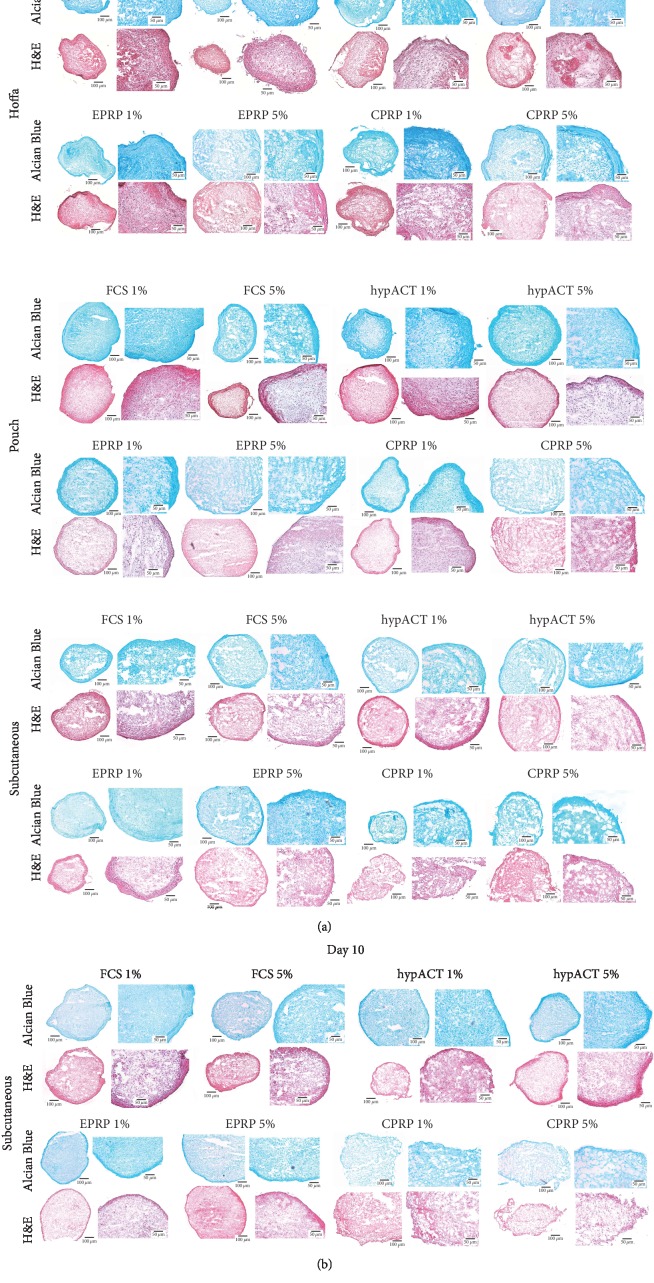
(a) Histology results from chondrogenic differentiation. CPRP=citrate platelet-rich plasma; EPRP=EDTA platelete-rich plasma; FCS=fetal calve serum; H&E=hematoxylin and eosin; hypACT=hyperacute serum. (b) Histology results—10-day differentiation of subcutaneous-derived cells. CPRP=citrate platelet-rich plasma; EPRP=EDTA platelete-rich plasma; FCS=fetal calve serum; H&E=hematoxylin and eosin; hypACT=hyperacute serum.

The histological cuts after chondrogenic differentiation are presented in Figures 5(a) and 5(b). These microscopic pictures show successful, chondrogenic differentiation.

In a qualitative assessment, pellet size is not different in between groups. Likewise, in a qualitative assessment, the histological appearance differs in between groups. Histological slices derived from Hoffa's fat pad pellets show a confined ECM morphology. Subcutaneous-derived samples show a diffuse and heterogenous ECM composition. Pouch fat-derived samples show a diffuse but homogenous ECM morphology. Many fissures and disintegrated tissue islands can be found in the extra-articular structure derived from subcutaneous fat. Furthermore, in groups with blood product concentrations of 1% the histological appearance of the ECM was confined and homogenous. Subcutaneous-derived samples were additionally cut after ten days of differentiation. This shorter differentiation period showed a confined and homogenous histological appearance of the ECM morphology.

#### 3.4.2. Osteogenic Differentiation

**Figure 6:**
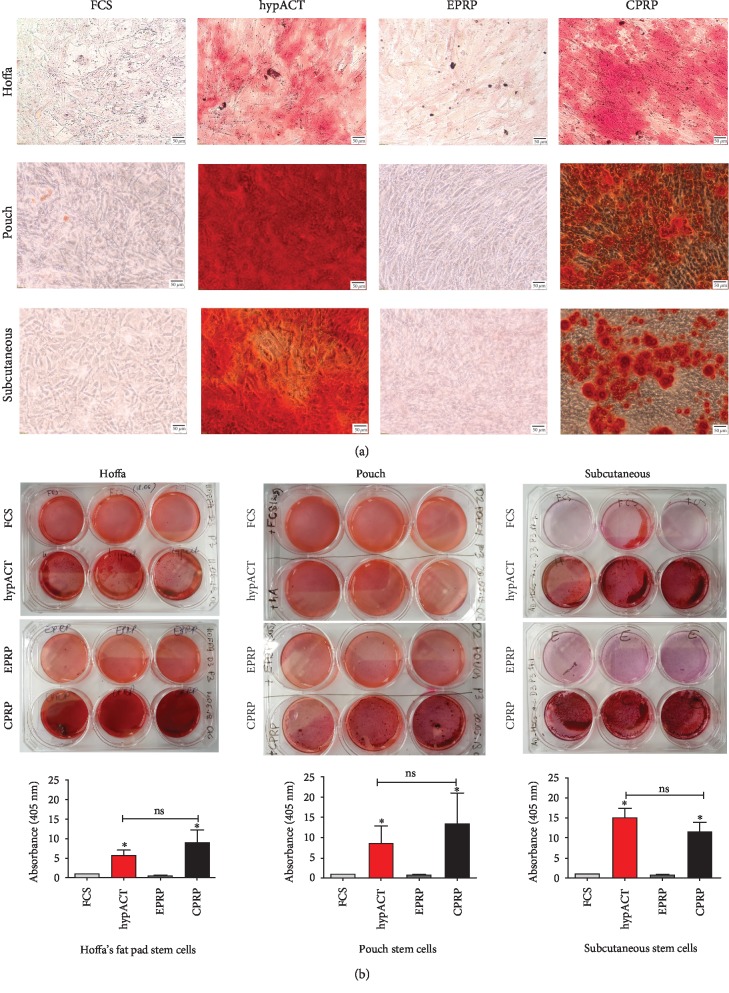
(a and b) Histology results from osteogenic differentiation. (a) Microscopic pictures. (b) Macroscopic pictures and dye extraction curves below. CPRP=citrate platelet-rich plasma; EPRP=EDTA platelete-rich plasma; FCS=fetal calve serum; hypACT=hyperacute serum; s.c.=subcutaneous. Results are expressed as the mean ± SD. ∗ represents *p* < 0.05, ∗∗ represents *p* < 0.01, and ∗∗∗ represents *p* < 0.001. Staining was performed after 21 days. Dye extraction results were normalized to the FCS group.

Alizarin Red staining showed the most intense mineralization in hypACT- and CPRP-supplemented groups independent of the harvesting site. In contrast, FCS- as well as EPRP-supplemented groups showed the least staining intensity amongst all stem cell populations. This finding was consistent in both macroscopic and microscopic picture analysis (Figures 6(a) and 6(b)). The quantification of the Alizarin Red accumulation corresponded with the microscopic observations (Figure 6(b)).

### 3.5. PCR—EPRP and hypACT Enhance Chondrogenic Gene Expression Whereas CPRP and hypACT Alike Enhance Osteogenic Gene Expression

#### 3.5.1. Chondrogenic

Before the analysis of gene expression, primers were designed and tested successfully regarding optimized temperature values for primer annealing.

COL2AB was not expressed in any of the three locations on day 0, which is why values after the three-week differentiation period are displayed in relative expression levels rather than in fold changes.

Differences in gene expression levels (typical chondrogenic-related genes: COL2AB and SOX9 and additional genes: MMP3 and COL1A1) were analyzed with regard to (i) harvest location, (ii) blood product supplementation, and (iii) blood product concentration.

Regarding location, a general observation for cells derived from intra-articular structures (Hoffa, pouch) reveals higher expression levels for typical chondrogenic genes (COL2AB, SOX9) in comparison to cells derived from the extra-articular structure of subcutaneous fat. Subcutaneous-derived cells showed no expression of COL2AB in any blood product group and inferior levels of SOX9 compared to intra-articular structures.

Regarding blood products, EPRP subgroups showed a significantly higher expression of COL2AB in Hoffa-derived cells. Moreover, EPRP subgroups showed a higher SOX9 fold change in cells from both intra-articular structures which were significant in the case of Hoffa's cells. CPRP showed the least expression of chondrogenic genes (SOX9, COL2AB) amongst all 3 blood product supplementations in all locations.

Regarding blood product concentration, EPRP1% leads to a significantly higher COL2AB expression in comparison to EPRP5% in Hoffa-derived cells. For SOX9 expression, EPRP5% leads to significantly higher expression levels in comparison to EPRP1%. CPRP5%, on the contrary, showed significant drops of SOX9 expression in comparison with CPRP1%. This finding was consistent both in pouch- and subcutaneous-derived cells. hypACT subgroups showed no significant differences in SOX9 expression for 1% versus 5% in all three locations.

The catabolic gene MMP3, essential in the breakdown of collagens, was expressed amongst all 3 locations on day 0 and significantly dropped after 3 weeks of differentiation. The only exemption was found in the subgroup CPRP5% for pouch-derived cells where MMP3 was significantly upregulated.

**Figure 7:**
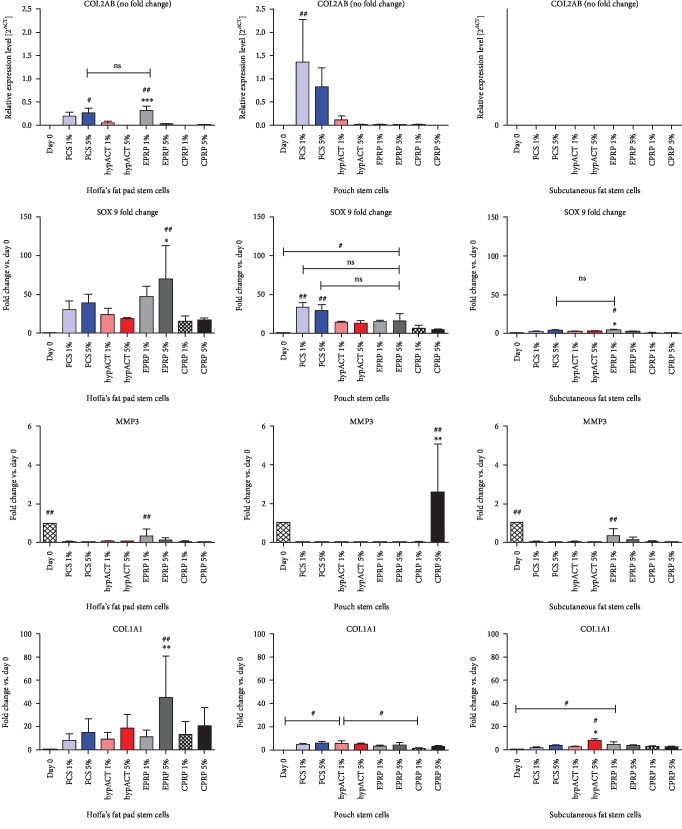
Gene expressions for chondrogenic differentiation experiments. COL1A1=collagen 1A1 gene; COL2AB=collagen 2 gene; CPRP=citrate platelet-rich plasma (black); EPRP=EDTA platelete-rich plasma (gray); FCS=fetal calve serum (blue); hypACT=hyperacute serum (red); MMP3=matrix metalloproteinase-3 gene. Results are expressed as the mean ± SD. # indicates a significant difference in gene expression between different treatment groups. # represents *p* < 0.05, ## represents *p* < 0.01, and ### represents *p* < 0.001. ∗ indicates a significant difference in gene expression within one treatment group. ∗∗ represents *p* < 0.01 and ∗∗∗ represents *p* < 0.001.

COL1A1 was expressed significantly higher with EPRP5% supplementation in Hoffa-derived cells compared to all other groups in this location. Likewise, hyperacute serum5% leads to significantly higher COL1A1 expression in subcutaneous-derived cells (Figure 7).

#### 3.5.2. Osteogenic

Genes specific for the different stages of osteogenic differentiation were analyzed during a 21-day differentiation period: OCN, ALPL, RUNX2, and COL1A1 (collagen 1).

OCN expression on day 0 tended to zero; therefore, OCN data are displayed in relative expression levels rather than in fold changes.

Overall, cells derived from Hoffa's fat pad showed the highest fold changes or relative expression levels, respectively.

The highest OCN expression was observed in the hypACT and CPRP groups in all three locations. The expression level was significantly higher within these two groups on day 21 compared to day 10 in cells derived from Hoffa's and subcutaneous tissues. Pouch-derived cells showed a minimal relative expression level overall.

ALPL fold changes dropped in the case of Hoffa-derived cells from day 10 to day 21. This drop was significant in this location for hypACT-, EPRP-, and CPRP-supplemented cells. The two other locations showed an overall less prominent fold change without significant differences.

RUNX2 showed a drop of fold change between day 10 and day 21 in the CPRP- and hypACT-supplemented cells both derived from Hoffa's and subcutaneous fat. This drop was significant for CPRP supplementation in Hoffa's cells.

**Figure 8:**
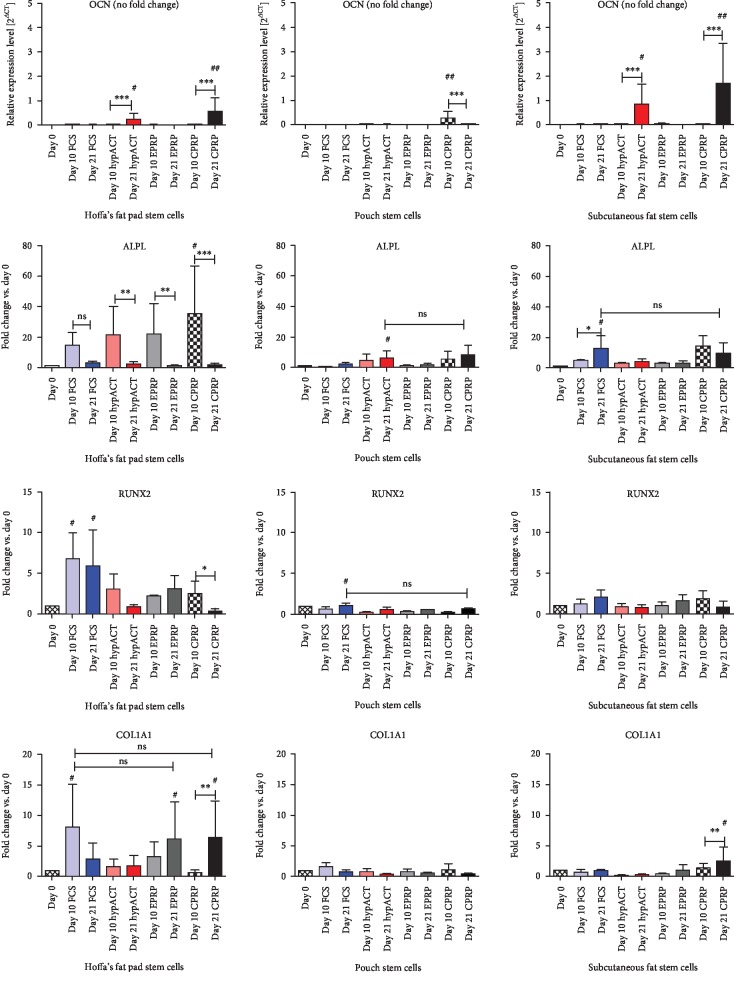
Gene expressions for osteogenic differentiation experiments. ALPL=alkaline phosphatase; COL1A1=collagen 1A1 gene; CPRP=citrate platelet-rich plasma (black); EPRP=EDTA platelete-rich plasma (gray); FCS=fetal calve serum (blue); hypACT=hyperacute serum (red); OCN=osteocalcin; RUNX2=runt-related transcription factor 2. Results are expressed as the mean ± SD. # indicates a significant difference in gene expression between the different treatment groups. # represents *p* < 0.05, ## represents *p* < 0.01, and ### represents *p* < 0.001. ∗ indicates a significant difference in gene expression within one treatment group. ∗∗ represents *p* < 0.01 and ∗∗∗ represents *p* < 0.001.

COL1A1 fold changes were the highest for CPRP and hypACT in Hoffa-derived cells on day 21. The increase from day 10 to day 21 was significant in the case of CPRP. Hoffa's cells supplemented with hypACT hardly showed any changes in between day 10 and day 21. Fold changes in the two other locations were comparably low. However, a significant increase from day 10 to day 21 was observed in subcutaneous cells supplemented with CPRP (Figure 8).

## 4. Discussion

The aim of this study was to demonstrate the differences in the osteogenic and chondrogenic differentiation potential of AD-MSCs derived from intra-articular versus extra-articular structures and to show the potentially beneficial effects of blood product supplementation to AD-MSC differentiation.

The process of stem cell isolation and cultivation is easier in comparison to primary chondrocytes or osteoblasts [38, 39]. The osteogenic and chondrogenic differentiation potential of MSCs in conjunction with the engrafting ability are crucial for cartilage [17, 40, 41] and bone regeneration in vivo [42]. Current literature describes examples of the beneficial effects of blood product supplementation on adipogenic [43], chondrogenic [44–46], and osteogenic differentiation of MSCs [45, 47]. Therefore, the rationale for this study was to enlarge the knowledge for the optimization of adipose stem cell treatment regimes combined with autologous blood products in clinical trials. Stem cells can be administered to the patient complying with the homologous use of the tissue to the defect site—the joint and intra-articular fat in this case—versus the currently applied nonhomologous use (subcutaneous fat). This approach could provide orthopaedic surgeons with the opportunity to use “surgical waste” as well as to skip regulatory hurdles for nonhomologous cell transplantation [25].

In this study, it was shown by flow cytometry that isolation of stem cells from both investigated intra-articular fat tissue sources—Hoffa's fat pad and the less-studied pouch fat as well as from subcutaneous fat—resulted in stem cell populations expressing typical stem cell surface markers.

The authors decided to include CD34 as a negative marker amongst others due to the recommendation of the position paper of the ISCT [35]. The rationale for stating CD34 to be a negative marker for MSCs is its known expression and therefore distinction to hematopoietic stem or progenitor cells as well as endothelial cells [35]. However, more recent literature suggests that CD34 may also be expressed by MSCs, as well as AD-MSCs [48–51]. More specifically, those papers show that CD34 positivity is gradually lost during culturing and plastic adherence [49]. CD34 positivity may be a “tissue-resident” cell feature only [50]. In conclusion, the authors followed the ISCT recommendations to use CD34 in a composite of negative MSC marker for two reasons:

- In many papers discussing the role of CD34, AD-MSCs were isolated from the so-called stromal-vascular-fractions and therefore harvested by liposuction or similar methods [48, 49]. These different harvesting and processing protocols may make a comparison to the results of this paper challenging with regard to immunophenotypic subpopulations
- The distinct role of CD34 for the tissue-forming capacity and biology remains poorly known [49]

Cryopreservation has to be taken into account as a limiting factor. P1 cells were either directly used after isolation or cryopreserved, thawed, and again cultured for further experiments as stated before. Cryopreservation has been shown to influence MSCs' therapeutic value with regard to impaired immunosuppressive activities, increased proapoptotic features, and impaired regenerative potential [52–54]. More recent studies show that MSCs do maintain their differentiation capacity and also immunomodulatory features after cryopreservation, but that a “reactivation” period of at least 24 h after thawing is beneficial [55]. Our P2 cells after thawing had more than 24 h to “reactivate” before experiments were started.

Passaging has also to be taken into account as a limiting factor. However, most recommendations for passaging MSCs for clinical use are for P3-5 [56]. Our experiments were performed with cells harvested only at P1 and P2 and therefore perfectly lie within the frame of recommended passages with respect to clinical use.

Another limiting factor may be intra-articular administration of drugs into donor's knees. Corticosteroids have been shown to decrease MSCs' viability and regenerative potential in a dose-dependent manner [57, 58]. Hyaluronan was shown to help maintain MSCs' stemness and to potentially (co-)stimulate chondrogenesis [59, 60]. These confounding factors need to be taken into account for the interpretation of the presented results. However, the above described potential effects may be less prominent due to the fact that patients received their last injection at least 6 months prior to TJR. Moreover, the concentration of the pharmaceutical agent in the joint can not exactly be evaluated but is likely to be diluted in the synovial fluid. Metabolic activity measured via XTT assay and supplementation with blood products were shown to have a stimulating effect likely supporting cell viability. This finding supports recommendations for the combined use of stem cell therapies along with blood products. This supplementation might increase the percentage of viable cells. The stem cells' proliferation seems to be important in cell-based therapies. Embryonic stem cells proliferate at high rates during development, whereas adult stem cells proliferate at a rather slow cycling in vivo [61]. Also, in the case of cartilage tissue, the regeneration process is based on stimulating the proliferation and matrix synthesis of neighbouring cells [62]. Cartilage does not contain blood vessels, therefore inflammation and fibrin clot formation cannot induce the healing. For that reason, boosting the proliferation of MSCs at the site of injury could accelerate the regeneration [63, 64]. This consideration elucidates the need for the enhancement of the MSC proliferation rate, while maintaining their particular phenotype [65]. In this study, we presented the stimulating effects of hyperacute serum and CPRP on AD-MSCs' metabolic activity. Linking these results with previous observations that MSCs' characteristic was retained after supplementation with hypACT and PRP, we may conclude that blood products have a beneficial effect on stem cell viability and their multipotency.

Current reports present divergent effects of platelet-rich plasma on chondrogenic differentiation [44, 66–69]; thus, this study is aimed at comparing the chondrogenic potential of different adipose stem cell sources combined with various blood products. A high-density 3D environment for stem cell chondrogenesis mimics interactions similar to those observed in precartilage condensations during embryonic development [70]; therefore, the 3D pellet cell culture model was established to evaluate the differentiation potential.

Based on the current literature, three specific markers for different stages of chondrogenesis were selected. The gene profiles for the early chondrogenesis stage are similar to those in precursor cells and include for example MMP3 and COL1 expression. In the later stages, cells show a progressive increase in markers of chondroprogenitors, which include SOX9, followed by that of mature chondrocytes expressing COL2 [71]. It was shown that cells derived from intra-articular structures, primarily Hoffa's fat pad, have a greater chondrogenic differentiation potential in comparison to extra-articular structures. Considering the expression level of SOX9 and COL2, stem cells isolated from Hoffa's fat pad reached a later stage of chondrogenesis compared to stem cells from pouch and subcutaneous fat. Based on both—the gene expression and glycosaminoglycan deposition in histological sections—hypACT along with EPRP were shown to further promote chondrogenesis. Surprisingly, EPRP—hardly used in clinical settings due to its known negative effects on cell viability [43, 72]—showed superiority regarding chondrogenesis in comparison to CPRP. A potential causative biological mechanism might be the chelating feature of EDTA, trapping free calcium ions which were already described to inhibit chondrogenesis [73]. Likewise, the “untrapped” free calcium ions in the case of CPRP might explain the inferior chondrogenic results both in histology as well as in gene expression. Also, the accumulation of platelets within chondrogenic pellets supplemented with CPRP might alter the forming tissue quality. Another biological mechanism might be the reported difference in growth factor concentration between CPRP and EPRP as well as hyperacute serum [24, 43]. It was reflected by different gene expression and glycosaminoglycan deposition favouring supplementation with EPRP1% rather than 5% which resulted in higher platelet accumulation within the pellet structure and lower ECM quality. Surprisingly, hyperacute serum concentration seemed not to have a significant influence on gene expression as well as on ECM formation within the pellets. According to the literature, growth factor concentration beyond critical levels may have serious side-effects [74, 75]. Graziani et al. showed that the use of concentrated PRP has an inhibitory effect on cell proliferation [75]. Broderick et al. and Harten and Svach observed that with a high level of TGF-*β*1 or VEGF, tissue formation was inhibited [76, 77].

An additional test concerning chondrogenic differentiation time was performed utilizing cells harvested from the subcutaneous fat which showed the least histological results. A shortened differentiation period was demonstrated to lead to superior histologic results. As clinically applied blood products are unlikely to stay in the joint over an extended period, this finding might be a supportive argument for clinical trial designs aiming for lower frequencies of application.

Shen et al. presented the different osteogenic potential of stem cells isolated from placenta tissues [78], proving that the selection of the stem cell source is an important step in stem cell therapy. Also, literature dealing with promoting effects of blood products upon osteogenesis is scanty. Arpornmaeklong et al. pointed out that a high concentration of PRP inhibited osteogenic differentiation [79]. Another study showed that PRP increased the proliferative capacity of osteoblast-like cells; however, the exact platelet concentration was not specified [80]. On the other hand, Kobayashi et al. reported the increased osteoblast migration and expression of osteogenic markers COL1, ALPL, and RUNX2, but no effects on osteoblast proliferation [81]. These examples reflect the problem of very divergent results of the PRP treatment. However, clinicians dealing with regenerative orthopedics need to treat bony defects, for example, in cases of subchondral bone lamella lesions, in order to sufficiently regenerate cartilage or to manage bone nonunions to give two examples.

Osteogenic differentiation is a complex process characterized by the expression of various osteogenesis-related genes, which level is time dependent during the following osteogenesis phases. RUNX2 is considered to be the central control gene for activating the program of osteoblastogenesis [82]. Studies confirm a high RUNX2 expression in the early stage of osteogenic induction and a significant drop during the differentiation into mature osteoblasts; moreover, RUNX2 is undetectable during the differentiation of osteoblasts into osteocytes [83, 84]. Numerous studies have indicated that ALPL may act as an early indicator of cellular activity and differentiation [85]. In the study of Ozawa et al., ALP gene expression of MSCs showed a peak level at day 12, which then decreased [86]. Zernik et al. also demonstrated that ALP activity is a very early marker of differentiation to the osteogenic lineage [87]. OCN appears immediately before the onset of mineralization. OCN levels increase with the progress of mineralization at the late stage in osteoblastic differentiation. Therefore, OC is a specific marker for mineralization and a good marker of osteoblastic phenotypes [86]. COL1 is an important component of the bone extracellular matrix; however, this protein cannot be considered bone specific since it can be identified in numerous unrelated cell types. Still, COL1 has been shown to play a role in the differentiation of the osteoblast phenotype [85].

In this study, the quantification of mineralization revealed that osteogenic differentiation was the most advanced in groups supplemented with CPRP and hypACT. The analysis of osteogenesis-related genes allowed estimating the progress of osteogenic differentiation between different blood product supplementation groups. CPRP and hypACT expressed superior levels of osteocalcin in comparison to EPRP and FCS. Additionally, the genes specific for earlier differentiation stages, ALPL and RUNX2, were shown to drop in expression from day 10 to day 21, especially in CPRP and hypACT groups and thereby provide further evidence for a promoting effect of blood products upon osteogenesis.

This different biological activity of EPRP and CPRP can be explained again by the chelating effects of EDTA, since it is known that the calcium ion content in the culture media can significantly increase osteogenic differentiation and mineralization [88].

This study is aimed at enlarging the basic knowledge about the differentiation potential of MSCs harvested from intra-articular versus extra-articular adipose tissue under blood product supplementation in order to drive advancement in novel AD-MSC-related therapeutic treatments. Limiting factors relativizing the presented findings might be the high age of donors, the small number of donors, and the challenging translation of in vitro findings towards in vivo therapies or trials. However, literature suggests that patient age is unlikely to be a limiting factor for the “regenerative potential” of harvested stem cells [30]. 3 locations per donor lead to 9 subgroups providing subtle ground for sound statistical analysis to support results. Also, the aim of this in vitro study was primarily to enlarge basic knowledge helping to design more sophisticated clinical trials rather than aiming for direct clinical translation.

## 5. Conclusion

The entire culture of expanded adipose-derived stem cells appeared differentiated to the desired lineage, on the basis of phenotypic characterization and histological analysis. Multiple lineages were not present.

In the case of hyperacute serum, the “natural” coagulation cascade is not delayed or stooped resulting in an autologous blood product containing no platelets and inferior levels of growth factors in comparison to platelet-rich plasma [24]. In conjunction with the abovementioned considerations for biological mechanisms, hypACT biochemical composition might be the reason for its comparably well-promoting effects both on chondrogenesis and osteogenesis unlike EPRP or CPRP only showing promoting effects for one distinct differentiation path.

The choice of anticoagulants in blood products seems to be of importance for MSC differentiation and thereby alters regenerative processes. Cell-free blood products without anticoagulants might be an alternative. Moreover, the concentration of blood products in reference to the total volume seems to be of importance for chondrogenesis, tending towards a preference for lower concentrations. This might be a relevant issue for clinical applications of blood products as the commonly used ones are hard to adjust in concentration. Moreover, the duration of blood product exposure seems to influence chondrogenesis, favouring shorter periods.

The presented findings investigate the biology of intra-articular AD-MSCs supplemented with autologous blood products in a preclinical setting. The aim is to enlarge the body of basic knowledge around AD-MSCs in order to codesign novel therapeutic approaches treating pathological conditions ranging from cartilage regeneration to osteoarthritis as well as bony defects.

## Figures and Tables

**Table 1:** Flow cytometric antibodies.

Positive markers	CD105 PerCP-Cy5.5/CD73 APC/CD90 FITC
Negative cocktail	CD45/CD34/CD11b/CD19/HLA-DR
Isotype control	mIgG1, *κ* PerCP-Cy5.5/mIgG1, *κ* APC/mIgG1, *κ* FITC (for positive markers) mIgG1, *κ* PE/mIgG2a, *κ* PE (for negative cocktail)

**Table 2:** Primers used in the real-time polymerase chain reaction.

	Amplicon (bp)	Annealing temp. (°C)
Osteogenic markers		
GAPDH	143	60
Forward: 5′-ACATCGCTCAGACACCATG-3′		
Reverse: 5′-TGTAGTTGAGGTCAATGAAGGG-3′		
ALPL	133	59
Forward: 5′-GATGTGGAGTATGAGAGTGACG-3′		
Reverse: 5′-GGTCAAGGGTCAGGAGTTC-3′		
RUNX2	148	61
Forward: 5′-TTCACCTTGACCATAACCGTC-3′		
Reverse: 5′-GGCGGTCAGAGAACAAACTAG-3′		
COL1A1	167	62
Forward: 5′-TCCAAACCACTGAAACCTCTG-3′		
Reverse: 5′-CCCCTGGAAAGAATGGAGATG-3′		
OCN	106	59
Forward: 5′-GGCGCTACCTGTATCAA TGG-3′		
Reverse: 5′-TCAGCCAACTCGTCACA GTC-3′		
Chondrogenic markers		
GAPDH	112	60
Forward: 5′-CTCTGCTCCTCCTGTTCGAC-3′		
Reverse: 5′-ACGACCAAATCCGTTGACTC-3′		
COL1A1	63	61
Forward: 5′-GGGATTCCCTGGACCTAAAG-3′		
Reverse: 5′-GGAACACCTCGCTCTCCAG-3′		
COL2AB	116	62
Forward: 5′-GCACCTGCAGAGACCTGA-3′		
Reverse: 5′-GGGTCAATCCAGTAGTCTCCAC-3′		
SOX9	73	59
Forward: 5′-TACCCGCACTTGCACAAC-3′		
Reverse: 5′-TCTCGCTCTCGTTCAGAAGTC-3′		
MMP3	91	58
Forward: 5′-CAAAACATATTTCTTTGTAGAGGACAA-3′		
Reverse: 5′-TTCAGCTATTTGCTTGGGAAA-3′		

**Table 3:** Cell count in blood products (RBC—red blood cells, WBC—white blood cells, PLT—platelets, and MPV—mean platelet volume).

	RBC (10^6^/*μ*l)	WBC (10^3^/*μ*l)	PLT (10^3^/*μ*l)	MPV (fl)
hypACT	0.03	0.18	3	6.5
EPRP	0.05	0.75	834	10.8
CPRP	0.04	1.23	505	9.8

## Data Availability

Not applicable (all the data are included).

## Abbreviations

3D: Three dimensional
AD-MSCs: Adipose-derived mesenchymal stem cells
ALPL: Alkaline phosphatase
ANOVA: One-way analysis of variance
BMA: Bone marrow aspirate
COL1: Collagen type 1
COL2AB: Collagen type 2
CPRP: PRP prepared in the presence of citrate
ECM: Extracellular matrix
EPRP: PRP prepared in the presence of EDTA
FCS: Standard fetal calf serum
GAPDH: Glyceraldehyde-3-phosphate dehydrogenase
hypACT: Hyperacute serum
ISCT: International Society for Cellular Therapy
MMP3: Matrix metalloproteinase-3
MPV: Mean platelet volume
MSCs: Mesenchymal stem or progenitor cells
OCN: Osteocalcin
P: Passage
PLT: Platelets
PRP: Platelet-rich plasma
qRT-PCR: RNA extraction and quantitative real-time polymerase chain reaction
RBC: Red blood cells
RUNX2: Runt-related transcription factor 2
SD: Standard deviation
SOX9: Transcription factor SOX9
TJR: Total joint replacement
TGF-*β*1: Transforming growth factor
VEGF: Vascular endothelial growth factor
WBC: White blood cells.
